# A lack of response of the financial behaviors of biodiversity conservation nonprofits to changing economic conditions

**DOI:** 10.1002/ece3.1281

**Published:** 2014-11-03

**Authors:** Eric R Larson, Alison G Boyer, Paul R Armsworth

**Affiliations:** Department of Ecology and Evolutionary Biology, University of TennesseeKnoxville, Tennessee, 37996-1610

**Keywords:** Adaptation, charity, conservation easements, financial ratios, land trust, nongovernmental organization

## Abstract

The effectiveness of conservation organizations is determined in part by how they adapt to changing conditions. Over the previous decade, economic conditions in the United States (US) showed marked variation including a period of rapid growth followed by a major recession. We examine how biodiversity conservation nonprofits in the US responded to these changes through their financial behaviors, focusing on a sample of 90 biodiversity conservation nonprofits and the largest individual organization (The Nature Conservancy; TNC). For the 90 sampled organizations, an analysis of financial ratios derived from tax return data revealed little response to economic conditions. Similarly, more detailed examination of conservation expenditures and land acquisition practices of TNC revealed only one significant relationship with economic conditions: TNC accepted a greater proportion of conservation easements as donated in more difficult economic conditions. Our results suggest that the financial behaviors of US biodiversity conservation nonprofits are unresponsive to economic conditions.

## Introduction

**Figure 1:**
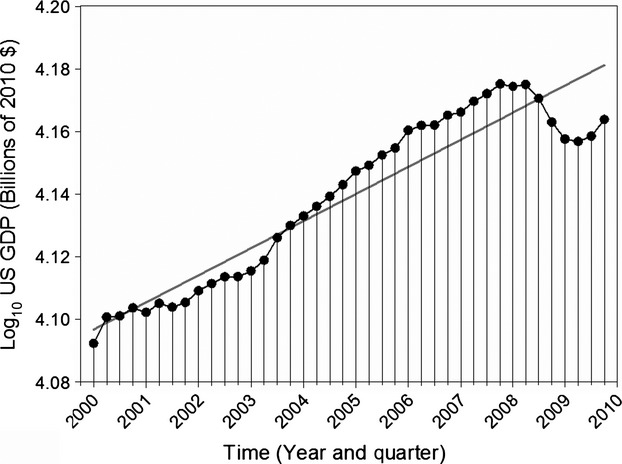
Log_10_ United States (US) gross domestic product (GDP) as billions of 2010 dollars ($) by quarter for 2000–2009 (black) with linear regression fit (gray).

The decade of the 2000s was characterized by highly variable economic conditions globally and within the United States (US), including a period of rapid growth followed by the largest recession since the Great Depression (Poole 2010; Fig.1). How these economic fluctuations have affected biodiversity conservation has been a subject of conjecture but little empirical evaluation. Some authors see opportunity to slow rates of habitat destruction and climate change during recessions and to decouple resumed economic growth from environmentally damaging production (Jackson 2009; Woodward 2009). Others caution that recessionary conditions may impair biodiversity conservation through diminished government revenues and related program cuts or by reduced charitable giving to nonprofit organizations (Bakker et al. 2010; Elliott 2011; Sayer et al. 2012).

These divergent predictions regarding the impact of changing economic conditions on conservation may hinge on conservation organizations' responsiveness or ability to adapt to change. The ability of conservation organizations to adapt to change has been suggested as a key driver of their overall effectiveness (Chapin et al. 2006 *;* Kenward et al. 2011), but has only recently begun to attract study (Brown et al. 2010; Jantarasami et al. 2010; Baral 2013). In contrast, responsiveness to changing conditions has long been an object of study in for-profit sectors (Carlsson 1989; Garvin 1993; Enlow and Katchova 2011). Economic theory suggests nonprofits may be less responsive than for-profits (Alchian and Demsetz 1972; Glaeser 2003), but empirical tests among nonprofit organizations remain scarce and primarily confined to sectors other than biodiversity conservation that have for-profit equivalents, such as health care (Duggan 2002; Malani et al. 2003).

Nonprofit organizations play an integral role in biodiversity conservation through activities including acquiring and restoring conservation lands and waters, providing environmental education, and seeking to influence government policies and their implementation through lobbying and litigation (Armsworth et al. 2012). Environmental nonprofits (of which biodiversity conservation nonprofits are a subset) have been one of the fastest growing segments of the overall US nonprofit sector in recent decades (Straughan and Pollak 2008). Yet biodiversity conservation nonprofits are reliant on revenue sources such as charitable donations, government grants, and foundation endowments that leave these organizations sensitive to economic fluctuations (Yen et al. 1997; Bakker et al. 2010). The biodiversity conservation nonprofit sector in the US is diverse, with organizations differing greatly in size and objectives (Armsworth et al. 2012), which may complicate characterizing responses to change. Yet, just as diverse organisms can evolve similar strategies to cope with highly variable “feasts and famines” of resource availability (McCue 2007; Armstrong and Schindler 2011), we expect that biodiversity conservation nonprofits may share some general financial behaviors for responding to economic booms and busts.

In this paper, we evaluate how economic conditions during the previous decade affected the financial behaviors of organizations in the biodiversity conservation nonprofit sector in the US. Our focus on financial behavior offers a tractable insight into organizational responses to economic events, although we recognize that bridging financial behavior to conservation effectiveness requires further study (see discussion). We use financial data from the US Internal Revenue Service (IRS) tax returns for a random sample of biodiversity conservation nonprofits to calculate “financial ratios” indicative of organization behavior. Originating from for-profit applications like predicting bankruptcy risk (e.g., Ohlson 1980), financial ratios have been applied to other nonprofit sectors to characterize organization behavior (Tuckman and Chang 1991; Trussel and Greenlee 2004; Keating et al. 2005; Zietlow 2010). Chabotar (1989) argued that “[financial] ratios are a much truer indicator of institutional priorities than any strategic plan.” Financial ratios have been widely used to demonstrate sensitivity of and responsiveness to economic conditions for a variety of for-profit firms and sectors (e.g., Youn and Gu 2010; Giordani et al. 2013). Accordingly, we anticipate that financial ratios may offer insights into common trends in the behaviors and management decisions made by biodiversity conservation nonprofits in response to changing economic conditions.

We also evaluate how economic conditions impacted the conservation tactics pursued by a single organization in more detail to complement our coarser analysis of cross-sectoral trends. We chose the largest biodiversity conservation nonprofit, The Nature Conservancy (TNC; Armsworth et al. 2012), focusing on TNC's conservation expenditures and land acquisition practices over the same decade. This case study analysis of the largest biodiversity conservation nonprofit allowed us to evaluate if findings of high or low responsiveness to changing economic conditions by financial ratios were consistent with more resolved tactical behaviors within an individual organization. Our emphasis on the financial behaviors of conservation organizations in response to changing economic conditions complements studies that instead seek to relate overall conservation activity to economic growth (Pergams et al. 2004; Fuentes 2011).

## Methods

### Cross-sectoral data

**Figure 2:**
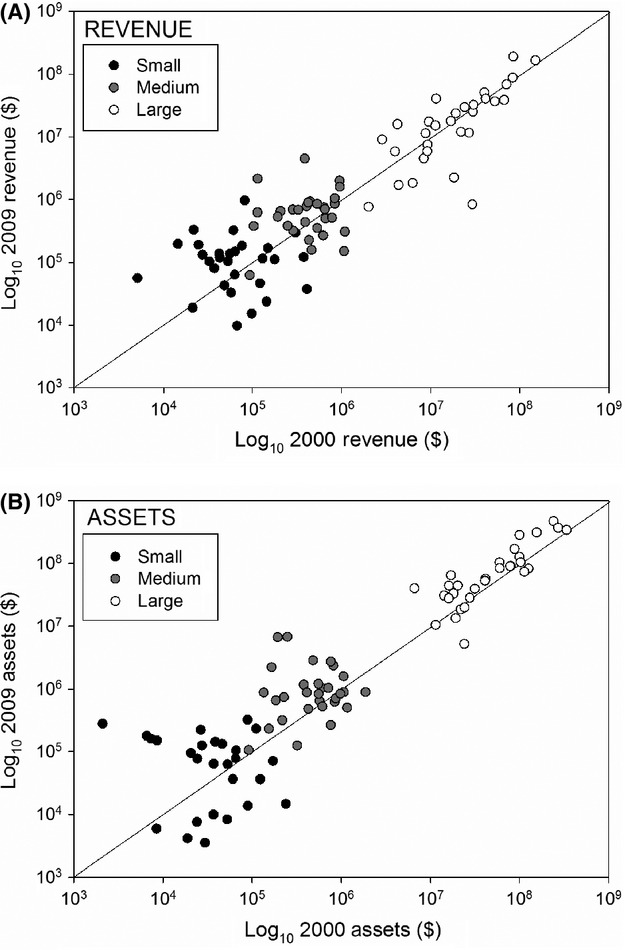
Inflation-corrected annual revenues and total assets for a random sample of 90 small, medium, and large biodiversity conservation nonprofits in 2000 and 2009 with a 1:1 line representing the boundary between negative and positive growth.

Our cross-sectoral analyses use a stratified random sample of 90 biodiversity conservation nonprofits drawn from an existing dataset of over 1700 such organizations (Armsworth et al. 2012). Sizes of biodiversity conservation nonprofits span six to seven orders of magnitude in the full dataset and are right skewed, with more small than large organizations. To ensure representation across this size gradient, we stratified the sample to contain 30 of the 200 smallest organizations, 30 from 200 around the median size, and 30 of the 200 largest organizations (Fig.2). For each nonprofit in the sample, we collected itemized data for reported revenues, expenditures, assets, and liabilities from their US tax returns for 2000–2009. Specifically, we used IRS 990 forms, which we accessed from the GuideStar website (http://www.GuideStar.org). We standardized all monetary amounts to 2010 US dollars ($) using the Consumer Price Index (http://www.bls.gov/CPI/). A minority of our nonprofits (35 of 90) reported in fiscal years different from the calendar year; for these organizations, we standardized fiscal years by calculating averaged monthly values and summing these into corrected calendar years.

The 90 organizations included are registered in 35 US states. They range across a diversity of conservation objectives and business models, from land trusts, to zoos, to advocacy groups for specific taxa, to institutes dedicated to basic and applied conservation research. In general, growth of these 90 organizations between 2000 and 2009 tracked that of US gross domestic product (GDP; Appendix 2). We included only organizations that filed IRS 990 forms every year between 2000 and 2009, excluding apparent exits (failures) for two reasons. Prior to 2008, small nonprofits with gross revenues below $25,000 were not required to file IRS 990 forms, and consequently true exits were difficult to parse from the more common incidence of organizations failing to file taxes for several consecutive years (Harrison and Laincz 2008). Further, environmental nonprofits have been reported to have exceptionally low exit rates relative to for-profit businesses (Harrison and Laincz 2008; Appendix 1).

We chose four financial ratios summarizing complementary aspects of nonprofit behavior: (1) liquid funds interval; (2) revenue concentration; (3) ratio of personnel costs to total expenditures; and (4) ratio of total liabilities to total assets (Table1). We used the liquid funds interval as an index of how many months a nonprofit could operate based on existing liquid assets (i.e., excluding land and buildings) if all incoming revenue ceased. We calculated the liquid funds interval as the ratio of total cash, savings, and investments relative to mean monthly expenditures. We anticipated that biodiversity conservation nonprofits should grow liquid funds under favorable economic conditions and deplete liquid funds during unfavorable economic conditions. For revenue concentration, we predicted that biodiversity conservation nonprofits might exploit more revenue sources under favorable economic conditions (something that requires active marketing and campaigns) and contract revenue sources under unfavorable economic conditions. Our ratio of revenue concentration scales from 1 (single revenue source) towards 0 (many revenue sources) calculated as the squared percentage share of each revenue source, of eight possible categories on IRS form 990, relative to total revenues (Tuckman and Chang 1991). We also included the ratio of expenditures specific to personnel (salaries, compensation, benefits) relative to total expenditures. Tuckman and Chang (1991) suggested that personnel or administrative costs offer a likely area for cuts during poor economic conditions. Finally, we included the ratio of total liabilities to total assets (following Trussel and Greenlee 2004) because we suspected that the willingness or ability to assume debts by biodiversity conservation nonprofits might vary under differing economic conditions in response to need (weathering a poor economy) or access (expanded or restricted lending pre- and post- 2007–2009 recession). Instances where liabilities greatly exceeded assets for a very small minority of organizations (seven) were reduced to values of one (liabilities equaling assets) to control the influence of extreme outliers. We log_10_ transformed liquid funds interval and arcsine square root transformed the remaining three ratios for all analyses.

**Table 1:** Financial ratios considered in cross-sectoral analyses with formulas for calculation and expected signs in response to increasing organizational size (log_10_ assets in 2000) and more favorable economic conditions (gross domestic product, GDP)

Financial ratio	Description	Formula	Organization size (assets)	Economic conditions (GDP)
Liquid funds interval	How many months could an organization operate if all incoming revenue ceased?	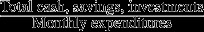	+	+
Revenue concentration	Is an organization reliant on one, few, or many revenue sources?	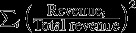	−	−
Personnel to total expenditures	What proportion of total expenditures does an organization spend on personnel?	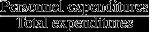	+	+
Liabilities to assets	What is the load of debts or liabilities an organization carries relative to all assets?	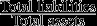	+	±

We emphasize that the coarse financial ratios outlined above do not represent all of the ways that an individual organization might respond to changing economic conditions. As one example, our measure of revenue concentration can express expansion of new or contraction of previous revenue sources through time. However, it does not differentiate between the identities of these revenue sources. An organizational transition from majority reliance on one revenue source (e.g., foundation giving) to another revenue source (e.g., government grants) of equal magnitude would go undetected (see Appendix 4). For this reason, we also sought to complement our use of coarse financial ratios with a more resolved analysis focusing on land acquisition practices of one major organization. However, we recognize that focused interviews of organizational leaders might be better suited for some aspects of more fine-grained responses (e.g., Mosley et al. 2012). As such, we also include some quotes from leaders of biodiversity conservation nonprofits on the sensitivity and responsiveness of their organizations to changing economic conditions (Appendix 4).

### The Nature Conservancy data

We complemented our cross-sectoral analyses with a more detailed analysis of the behavior of the largest nonprofit in US biodiversity conservation, TNC. TNC manages 16% of total revenues and 25% of total assets reported by the sample of 1700 US biodiversity conservation nonprofits examined by Armsworth et al. (2012). As TNC is primarily a land trust, we analyzed this organization's patterns of land acquisitions in the lower 48 US states between 2000 and 2009. We included lands acquired as fee simple (*n*  = 4333) and using conservation easements (legal agreements restricting land uses by private owners; *n*  = 1451). For each transaction, we considered the total area (hectares), total cost ($ US 2010 equivalent), the proportion of costs that were donated relative to fair market values estimated from independent appraisals of property values, the price per hectare of acquisitions, and finally the ratio of conservation easements to fee simple acquisitions. We aggregated these fields across deals done in each financial quarter in 2000 through 2009. All TNC responses were log_10_ transformed for analyses, with additional detail on sources and management of TNC data given by Fishburn et al. (2013) and Davies et al. (2010).

### Data analyses

We sought to relate our indicators of nonprofit behavior (above) to changing economic conditions. We used log_10_ transformed US GDP in billions of 2010 $ (Fig.1) as our predictor of economic conditions. We chose to use GDP over other measures like stock market indices (Pergams et al. 2004) because we felt that GDP would be most relevant to the breadth of biodiversity conservation nonprofits included in our cross-sectoral analyses. As sensitivity tests, we also evaluated organizational responses to changes in their own revenues from year to year and the effect of revenue growth on financial behaviors (Appendix 3). To account for the confounding of GDP with time (Fig.1), we performed regression analyses with the behavioral response indicators and GDP linearly detrended by time (i.e., residuals). We also included in our models either nonprofit size (cross-sectoral analyses) or financial quarter (TNC analyses). Quarter was included as a predictor for TNC because preliminary data investigation revealed a potential effect of quarter on land acquisition activities owing to either a preference by buyer (TNC) or sellers for fourth quarter transactions. For cross-sectoral analyses, organization size was included in models as the log_10_ transformed assets of each biodiversity conservation nonprofit at the beginning of our study time period (Fig.2), with organization identity incorporated as a random effect in linear mixed models (*nlme* library, R). We also included a set of models that incorporated an interaction term between biodiversity conservation nonprofit size and economic conditions (time-detrended GDP). Predictions of the role of organization size on financial ratio responses are given by Table1 (see also Tuckman and Chang 1991; Trussel and Greenlee 2004). Pseudo- *R* ^2^ values were calculated for cross-sectoral mixed models as the relationship of model fitted to observed response values.

## Results

Three of the four financial ratios considered in cross-sectoral analyses were affected by biodiversity conservation nonprofit size (Table2). Specifically, larger organizations are characterized by having more liquid assets, more diverse revenue concentration (a lower value by our index), and higher personnel costs proportional to total expenditures (Table2). The direction of these relationships is consistent with the expectation that smaller nonprofits are more financially vulnerable than larger nonprofits (Table1). The ratio of liabilities relative to total assets was not affected by organization size.

**Table 2:** Results of linear regression models for financial ratios of 90 biodiversity conservation nonprofits after detrending each response and gross domestic product (GDP) by time, and including organization as a random effect. Results are given for models excluding and including a term for interaction between nonprofit size and GDP

	Size (SE)	GDP (SE)	Size × GDP (SE)	Pseudo- *R* ^2^
No Interaction				
Liquid funds interval	0.181 (0.030)***	2.968 (1.211)*	–	0.206
Revenue concentration	−0.088 (0.024)**	−2.255 (0.780)**	–	0.100
Personnel to total expenditures	0.042 (0.015)**	−1.479 (0.478)**	–	0.062
Liabilities to assets	0.017 (0.017)	0.584 (0.737)	–	0.007
Interaction				
Liquid funds interval	0.181 (0.030)***	8.093 (5.230)	−0.866 (0.860)	0.206
Revenue concentration	−0.088 (0.024)***	11.634 (3.334)***	−2.347 (0.548)***	0.106
Personnel to total expenditures	0.042 (0.015)**	−4.899 (2.063)*	0.578 (0.339)	0.063
Liabilities to assets	0.017 (0.017)	−1.130 (3.186)	0.290 (0.524)	0.007

Significance of coefficients is given as ≤0.001 (***), ≤0.01 (**), and ≤0.05 (*). Pseudo- *R* 2 is given as the relationship of model fitted to observed response values.

Specific to our focal question, two of four financial ratios (liquid funds index, revenue concentration) responded as predicted (Table1) to economic conditions (Table2). Further, the significant interaction of organization size and economic conditions reveals that large biodiversity conservation nonprofits experienced the most severe increases in revenue concentration under worsening economic conditions, as smaller organizations were characterized by concentrated revenues regardless of economic conditions (Table2). Interestingly, the ratio of personnel costs to total expenditures was significant but in the opposite direction hypothesized (Table1). Personnel costs became a larger component of total expenditures under worsening economic conditions (Table2; but see Appendix 3). The ratio of liabilities to assets was altogether unresponsive to changing economic conditions. Despite some significant coefficients for economic conditions on financial ratio responses, our low pseudo- *R* ^2^ values suggest that biodiversity conservation nonprofits are not particularly responsive to changing economic conditions (Table2).

Our analysis of TNC's land acquisition behavior provides an opportunity to test for behavioral responses to economic conditions at a much more resolved, if organization specific, level. However, again we detected little discernable response in behavior. Only one TNC land acquisition behavior was significantly affected by GDP and explained a meaningful proportion of the variance (Table3). The proportion of conservation easement costs that TNC accepted as donated relative to appraised fair market values increased under poor economic conditions and decreased under good economic conditions (Table3). As anticipated by our preliminary data explorations, quarter affected many TNC behaviors, with fourth quarter preferences for easement deal size as measured by area, the proportion of easements to fee simple acquisitions, and the proportion of deals that were donated (Table3).

**Table 3:** Results of linear regression models for TNC land acquisition responses after detrending each response and gross domestic product (GDP) by time, given as totals and specific to either fee simple acquisitions or conservation easements

	Quarter (SE)	GDP (SE)	*R*^2^
Deal size ($)	0.043 (0.036)	3.418 (4.591)	0.050
Fee simple acquisitions	0.045 (0.038)	2.449 (4.838)	0.042
Conservation easements	0.049 (0.051)	10.319 (6.536)	0.082
Deal size (Hectares)	0.085 (0.042)	3.374 (5.328)	0.107
Fee simple acquisitions	0.060 (0.051)	−1.423 (6.429)	0.038
Conservation easements	0.154 (0.061)*	11.628 (7.723)	0.187
Easements: fee simple acquisitions	0.078 (0.035)*	1.187 (4.509)	0.116
Proportion donated	0.168 (0.035)***	−3.137 (4.459)	0.392
Fee simple acquisitions	0.147 (0.041)**	0.316 (5.279)	0.253
Conservation easements	0.106 (0.026)***	−6.671 (3.268)*	0.369
Cost ($) per hectare	−0.041 (0.030)	0.044 (3.833)	0.048
Fee simple acquisitions	−0.015 (0.030)	3.872 (3.826)	0.034
Conservation easements	−0.105 (0.050)*	−1.309 (6.343)	0.108

Significance of coefficients is given as ≤0.001 (***), ≤0.01 (**), and ≤0.05 (*).

## Discussion

Scientists regularly express in popular media (e.g. *,* Woodward 2009) or as asides in scientific manuscripts (e.g., Bakker et al. 2010) the belief that economic events like recessions can harm or help the cause of biodiversity conservation, yet almost no studies have quantified relationships between economic conditions and conservation activity (but see Pergams et al. 2004; Elliott 2011). We propose that the effect of economic fluctuations on biodiversity conservation will be determined in part by how conservation organizations buffer themselves against and respond to change. We provide the first empirical investigation into the effects of changing economic conditions on the financial behavior of biodiversity conservation nonprofits. We found that few measures of financial behavior were meaningfully affected by economic conditions whether evaluated for a cross-sectoral sample or the largest individual organization.

There are growing calls to examine the capacity of conservation organizations to adapt to changing conditions (West et al. 2009; Barbour and Kueppers 2012), but empirical investigations of this adaptive capacity remain scarce (but see Baral 2013). Our results suggest biodiversity conservation nonprofits may have little adaptive capacity, at least with regards to changing economic conditions. Alternatively, funding processes and conservation activities may operate on too long of time lags for our methods to detect behavioral responses to changing economic conditions. For example, land trusts like TNC often negotiate transactions with private landowners and cost-sharing government partners over many years, potentially obscuring evidence of responsiveness to current economic conditions (Appendix 4). List (2011) similarly observed that charitable giving to the nonprofit sector is asymmetrical with respect to economic conditions: good economic conditions correspond with increased charitable giving to a greater extent than poor economic conditions correspond with reduced giving, likely because revenue is often tied to contracts agreed upon years in advance.

When looking across the sector, we found that organizations may grow liquid funds under favorable economic conditions and deplete them under unfavorable economic conditions. We also found that revenue concentration, particularly for larger organizations, expands and contracts inversely with economic growth. However, contradicting our predictions and those of past work on financial vulnerability in nonprofits (e.g., Tuckman and Chang 1991), we were surprised to find that biodiversity conservation nonprofits may preferentially protect personnel when economic conditions are poor, likely at the cost of program activities (but see Appendix 3). Yet the variance explained by the models remained low, and the prevailing signal was one of little discernible behavioral response to changing economic conditions. A different approach might have focused on expenditures (e.g., diversity of programmatic offerings) rather than revenues and assets. Lowry (1997) tested for such effects of economic conditions on expenditures (i.e., spending on public goods vs. fundraising incentives) by 16 environmental nonprofits in the 1990s. Consistent with our results, Lowry (1997) found no evidence that external conditions impacted behavior of these organizations.

Land acquisition activities by TNC provide a more direct measure of on-the-ground conservation behavior, but were also not particularly responsive to GDP. However, the one significant exception does provide an interesting demonstration of the potential interaction between economic conditions and behavior of biodiversity conservation nonprofits. TNC accepted a greater proportion of conservation easements as donated in less favorable economic conditions relative to good economic conditions. This result, especially when put alongside a lack of response in the overall amount of conservation activity (e.g., easements acquired whether by cash or area), suggests TNC maintains their pace of conservation activity under poor economic conditions in part by taking as donations lands they might not prefer under more favorable conditions. This behavior also likely displaces some of the cost of conservation onto state and federal governments via land owner tax deductions for easement donations at times (economic recessions) when government budgets are already stressed by decreases in revenue.

Surveys and interviews of employees or board members might be used to test our conclusion of little responsiveness by biodiversity conservation nonprofits to economic conditions, and also to further characterize how such responsiveness relates to meeting organization objectives and conservation goals (e.g., Brown et al. 2010; Jantarasami et al. 2010). Mosley et al. (2012) used such surveys to evaluate adaptive tactics of human services nonprofits to economic recessions, and found results largely consistent with our study: larger organizations had more overall capacity for responsiveness, but most nonprofits exhibited little responsiveness to changing economic conditions. To provide additional context to our analyses, we report brief quotes from a small selection of leaders (executive directors, board members, etc.) of biodiversity conservation nonprofits on how economic conditions affect their organizations and how they respond (Appendix 4). These comments reflect a breadth of ways that the economy has (or has not) affected these organizations and the diversity of their financial responses, whether strategic or opportunistic. Some biodiversity conservation nonprofits “…just got bigger and bigger…” through the recent recession while others “…proactively down-sized…”, and some organizations have “… nothing built into our by-laws to take economic conditions into account…” whereas others “…approach these issues pretty strategically…” (Appendix 4).

An alternative interpretation of our results might conclude that many biodiversity conservation nonprofits simply do not prioritize responsiveness to changing economic conditions as an organizational objective. Such an interpretation might instead argue that many organizations seek to simply balance expenditures to revenues from year to year while maintaining other financial attributes (e.g., liquid funds interval) in some kind of consistent “fiscal homeostasis.” Related, Zietlow (2010) in a study of religious nonprofits in the US under recessionary conditions categorized four financial paradigms for these organizations, ranging from those seeking to just meet or slightly exceed their budgets on one end of a gradient to those aspiring to high financial flexibility on the other. Similar to our perspective, Zietlow (2010) characterized those nonprofits not managing for financial flexibility or responsiveness as “muddling through” or only aspiring to survival at best, often because these organizations were constrained by a “current services” trap that led to underinvesting in their own financial flexibility or liquidity. We believe an argument that biodiversity conservation nonprofits should not emphasize financial responsiveness to changing economic conditions is similar: that the mission of immediate biodiversity conservation is so urgent that organizations should not manage their finances for future contingencies or flexibility. Our interviews with biodiversity conservation nonprofit leaders (Appendix 4) do reveal gradients of intended or desired financial responsiveness, and we recognize that adaptation to changing economic conditions may not be a priority for some of these organizations. Whether it should be – and what that means for biodiversity itself – is a topic that our study invites more inquiry into.

We conclude by emphasizing that efforts to characterize effectiveness of conservation activity for the sector in aggregate remain in their infancy (Gaston et al. 2006). As others have noted (e.g., Chabotar 1989), financial ratios provide one method for examining quantitatively the behaviors of very diverse nonprofit organizations in response to shared events (e.g., recessions). Given the important role of nonprofits in biodiversity conservation, we have considerable and important knowledge gaps in understanding, and perhaps enhancing, their responsiveness and adaptability to change. At a minimum, we hope our work introduces new tools (i.e., financial ratios), observations, and hypotheses to inspire and inform subsequent studies on responsiveness of biodiversity conservation nonprofits to changing conditions.

## APPENDIX

### Appendix

Our focus in the main text is on seeking evidence of financial behavioral responses by biodiversity conservation nonprofits to changing economic conditions. However, to contextualize our study relative to other work on the nonprofit sector and economic conditions, we summarize here some additional results on exit rates of our cross-sectoral sample of biodiversity conservation nonprofits (see Harrison and Laincz 2008; Appendix 1) and on growth of these organizations (see Pergams et al. 2004; Straughan and Pollak 2008; Armsworth et al. 2012; Appendix 2). We also evaluate whether biodiversity conservation nonprofits are more responsive to changes in their own revenues rather than changes in overall economic conditions (GDP), as well as whether longer term organizational growth in revenues affects financial behavior (Appendix 3). Finally, we report results of interviews with a small selection of biodiversity conservation nonprofit leaders to provide further texture and context on the ways that these organizations respond to changing economic conditions (Appendix 4).

### Appendix 1

#### Exit rates of biodiversity conservation nonprofits

Nonprofit organizations are thought to have low exit rates relative to for-profits. For example, nonprofits cannot redistribute earnings or assets as profits at liquidation, raising the decision threshold to exit above that to declare bankruptcy in for-profit businesses (Harrison and Laincz 2008). Harrison and Laincz (2008) reported mean annual exit rates of only 2.1% for approximately 290,000 nonprofit organizations in the US between 1989 and 2000, and exit rates of only 2.3% specific to environmental nonprofits (of which biodiversity conservation nonprofits are a subset). For this reason, as well as difficulty in identifying true exits from neglect to file IRS tax returns (see main text), we anticipate minimal effect of organization exits as sample selection bias in our analysis of biodiversity conservation nonprofits behaviors to changing economic conditions. However, cross-sectoral analyses seeking to identify predictors of biodiversity conservation nonprofit failure (exits) due to organizational behavior or economic conditions would be an interesting area of further study. At a minimum, our analysis evaluates behavioral responses to changing economic conditions for a sample of biodiversity conservation nonprofits that were robust to (avoided) failure between the years 2000 to 2009.

To compile our cross-sectoral sample of biodiversity conservation nonprofits, we randomly sampled 600 organizations drawn from a larger set of 1700 organizations considered in Armsworth et al. (2012), which were partitioned as 200 of the smallest, 200 around the median size, and 200 of the largest organizations. We worked sequentially until arriving at a stratified random sample of 90 organizations (30 each in small, medium, and large categories) for which IRS tax returns for all years between 2000 and 2009 were available or could be acquired. A large number of organizations had missing forms for at least some years; 145 organizations were evaluated before arriving at 90 with complete forms for the years considered. Effort was made to acquire tax forms for nearly all organizations with missing years; 31 biodiversity conservation nonprofits were contacted by mail, and 29 were contacted by e-mail soliciting for missing forms. Seven of these replied with requested forms. Many nonresponses appeared to be just that: nonresponses from busy but extant organizations. However, we identified six of 64 small organizations (9.4%), two of 41 medium organizations (4.9%), and zero of 40 large organizations that may have failed or exited the sector between 2000 and 2009 (i.e., no mail or e-mail response provided and websites absent or inactive).

Over our study decade, our total exit rates (above) translate to a mean annual exit rate of 0.6% partitioned as 0.9% for small organizations, 0.5% for medium organizations, and 0.0% for large organizations. These values are even lower than mean annual exit rates reported for nonprofits, and environmental nonprofits specifically, by Harrison and Laincz (2008). One difference between our two studies was that our smaller sample size allowed for investigation of actual failure or exit in cases where tax returns were missing (above), whereas the much larger dataset of Harrison and Laincz (2008) necessitated defining exits as any instance in which an organization did not file tax returns in any remaining year of the time sequence (e.g., an organization that did not file in year *t * + * * 1 was not counted as an exit if it filed in year *t * + * * 2, *t * + * * 3, etc.). As such, Harrison and Laincz (2008) note that they “are most likely presenting an *overestimate* of exit,” particularly for later years in their study in which less time was available to distinguish true exits from IRS noncompliance.

We performed a sensitivity test to evaluate potential effects of sample selection bias on our parameter estimates (Table2). We simulated organization exits at our observed rate (above) in a biased manner to exclude those organizations from our sample of 90 anticipated as most vulnerable to failure. We excluded 9.4% of our 30 small organizations (three total), 4.9% of our 30 medium organizations (two total), and none of our large organizations. Excluded organizations had the largest observed reductions in assets between 2000 and 2009 (Fig.2) to represent those biodiversity conservation nonprofits under the most severe financial stress and at greatest risk of failure (Trussel and Greenlee 2004). We then used the influence ME library in R to exclude these five organizations from the same linear mixed effects regression models as applied in our main analysis (Table2), and compared parameter estimates and standard errors between the two analyses (Table A1). In all cases, parameter estimates and standard errors after omitting a biased simulation of organization exit closely resembled those from our full analysis (Table A1).

### Appendix 2

#### Growth of biodiversity conservation nonprofits

We report here growth by revenues and assets for our sample of biodiversity conservation nonprofits between 2000 and 2009. We test for significant differences in geometric mean growth rates between organizational size categories (Fig.2) using Kruskal–Wallis rank order tests and against mean geometric growth in US GDP over this decade with single sample *t* -tests.

The 90 biodiversity conservation nonprofits analyzed experienced little growth in annual revenues from the years 2000 to 2009, with a mean value of 0.1% that did not vary significantly (*H*_2_ = 2.873, *P*  = 0.238) between small (−1.4%), medium (4.4%), and large(−2.5%) organizations. Growth in revenues did not differ from overall growth in US GDP (3.8%) for small (*t*  = −0.906, *P*  = 0.372) and medium organizations (*t*  = 0.287, *P*  = 0.776) but was significantly lower for large organizations (*t*  = −2.890, *P*  = 0.007).

Biodiversity conservation nonprofits saw consistent increases in assets over this time period with a mean growth rate of 6.4% (Fig.2), which also did not vary significantly (*H*_2_ = 0.676, *P*  = 0.713) between small (7.5%), medium (8.1%), and large (3.7%) organizations. No organizational size category experienced growth in assets significantly different from that of US GDP (*t* 's = −0.072–1.629, *P* 's = 0.114–0.944). As such, performance by growth for our individual US biodiversity conservation nonprofits generally matched growth of the US economy as GDP between 2000 and 2009. The sector as a whole still may have experienced growth in excess of GDP if the number of nonprofits itself increased due to a high rate of organizational entrance and low rate of organizational exits as suggested between 1989 and 2000 by Harrison and Laincz (2008).

### Appendix 3

#### Organizational revenue and financial behavior

We sought to evaluate how biodiversity conservation nonprofits adjust their financial behaviors in response to economic events such as periods of widespread growth or recessions. One reason we failed to find pronounced responsiveness of these organization to economic trends, as represented by GDP, may be that they are more responsive to organization-specific events, as represented by changes in their own revenue. To evaluate this possibility, we performed sensitivity tests in which our original analyses for the cross-sectoral data were repeated by (1) substituting time-detrended (i.e., residual) revenue for each organization in place of GDP and (2) repeating the original analyses with GDP but including geometric growth in revenue (see above) for each organization. The first of these sensitivity tests sought to evaluate whether organizations were more responsive in their financial behaviors to their own year to year revenue patterns than broader economic conditions. The second of these sensitivity tests sought to evaluate whether including information on trend in organizational revenue, whether growing or shrinking over the 2000–2009 time period, was reflected in financial behaviors. It might be expected that an organization reliably growing in revenue during poor economic conditions may appear unresponsive to economic conditions, whereas an organization shrinking in revenue might exhibit financial behaviors that appear counterintuitive relative to a growing economy.

We found no evidence that biodiversity conservation nonprofits were more responsive in their financial behaviors to their own revenues than to the overall economy. Substituting revenue for GDP in our analyses produced models with performance equivalent to that reported in the main text (Table A2). Revenue did not significantly affect any of our financial ratios with the exception of the ratio of personnel to total expenditures in the model that included an interaction term with organization size (Table A2). Interestingly, this result both complied with our prediction of how organizations should behave in response to financial stress (Table1) and contradicted the result we found for financial behaviors in response to GDP (Table2). Biodiversity conservation nonprofits may be more responsive in managing personnel costs relative to total expenditures in reaction to their own revenue trends than in reaction to overriding economic conditions. This responsiveness may be more pronounced in larger rather than small organizations per the significant interaction term (Table A2), a result likely explained by larger organizations having a greater proportion of personnel expenses relative to total expenditures to selectively grow or cut in response to financial conditions (Table2). In no cases where trend in revenue (geometric revenue growth; see above) was included in models incorporating GDP were coefficients for this variable significant (Table A3). Per the results in the main text, low pseudo- *R* ^2^ values suggest that biodiversity conservation nonprofits are not particularly responsive in their financial behaviors to changing conditions, regardless of whether these changing conditions are organization-specific revenue streams or overriding economic conditions.

### Appendix 4

#### Interviews with biodiversity conservation nonprofit leaders

We appreciate that financial ratio analysis may not be intuitive to many practicing conservation scientists and managers. What specifically do our predictions and results from financial ratio analysis mean? Are they realistic, and do they represent the challenges organizations face and the decisions they actually make? Financial ratio analysis has been advocated as an empirical and objective measure of what organizations do; its lack of dependence on potentially subjective opinion is touted as one of its strengths (Chabotar 1989). Further, financial ratio analysis also standardizes the measure of responsiveness between all organizations by drawing from financial reporting on IRS tax returns. However, these benefits come with tradeoffs of resolution and specificity. As an example outlined in our main text, the ratio of revenue concentration we used (adapted from Tuckman and Chang 1991) can represent expansion or contraction of revenue sources in a funding portfolio, but does not differentiate between identities of these revenue sources. Consequently, a transition from diminished reliance on one revenue source to proportionally increasing reliance on another revenue source over a period of economic change might go undetected.

More resolved behavioral responses of biodiversity conservation nonprofits to changing economic conditions could be investigated a variety of ways. In the main text, we analyzed land acquisition practices of a major biodiversity conservation nonprofit, The Nature Conservancy (TNC), as a means of giving more detail on how a single organization responded to changing economic conditions. This analysis largely supported our findings of low responsiveness of biodiversity conservation nonprofits to changing economic conditions as observed from financial ratios. Alternatively, some researchers have used interviews with nonprofit members and leaders to explore patterns of responsiveness and identify specific actions taken in response to events such as economic recessions (e.g., Mosley et al. 2012). Such interviews have been applied to evaluate the responsiveness or adaptability of conservation organizations to perturbations like climate change (Brown et al. 2010; Jantarasami et al. 2010).

Accordingly, we supplement our main text here with a small selection of commentary and quotes from leaders in biodiversity conservation nonprofits, who we spoke to following completion of our study. We interviewed five leaders associated with biodiversity conservation nonprofits across a range of missions and sizes. Interviewed individuals included a CEO from a regional organization focused on land protection and wildlife conservation (Individual A), a committee member from a local organization focused on funding biodiversity conservation research (Individual B), a board member from a local land trust (Individual C), a member of the board of directors of a regional land trust (Individual D), and a chair of a large, general international conservation organization (Individual E). Individual A is affiliated with an organization located outside the US, although we anticipate they share general patterns of financial decision-making with US biodiversity conservation nonprofits and have experienced recent (i.e., recessionary) economic conditions on similar timescales. Individuals B, C, and D and their organizations are located in three different US states in disparate geographic regions.

We introduced the questions, analyses, results, and conclusions from our research in advance to individuals A–E, and invited open responses on how their organizations are affected by economic conditions (if at all) and how they respond to changing economic conditions (whether opportunistically or strategically). We emphasize that these conversations do not represent the kind of rigorous qualitative studies often used to pursue similar questions (e.g., Mosley et al. 2012). Instead, these interviews serve as a means to provide texture and context from personal experiences on how biodiversity conservation nonprofits respond to changing economic conditions. We have edited together quotes from the leaders identified anonymously above, in a sequence that runs from whether or not organizations are affected by the economy (Appendix 4a) to how they respond, both opportunistically and strategically (Appendix 4b). These quotes are interspersed with our own brief commentary and connections to our findings.

### Appendix 4a

We begin with comments on how changing economic conditions affect these organizations, including two observations that economic effects could be minor or nonexistent in some cases:

Individual A (1): Our overall growth trajectory from 2002 to about 2013 was pretty consistent. We just got bigger and bigger. Parts of our region are relatively prosperous, so we are somewhat insulated from the recession in terms of individual giving. Our membership is mostly made up of people with higher wages, and they just were not affected by the recession in the way that people with lower incomes were. Individual E (1): We proactively downsized in response to the recession in case we could not continue to raise funds. But we actually grew right through the recession, counter intuitively. This was mostly due to government money or stimulus money, which was on multiple year grants, and we really did not miss a beat.

These examples highlight that changing economic conditions, such as the 2007–2009 financial crisis, should not necessarily be anticipated to affect all organizations similarly (i.e., these organizations grew despite the recession). Specific to biodiversity conservation nonprofits, our own data show that some organizations grew between 2000 and 2009 at a pace exceeding GDP, whereas other organizations decreased in size, but in general growth of our sample organizations resembled that of GDP (see above and Fig.2). We suggest that variation in growth between biodiversity conservation nonprofits over the same economic conditions supports our decision to also perform our financial ratio analysis as responses to organization-specific revenue patterns (see above) rather than only on GDP — a sensitivity test that did not overturn our main text conclusions of limited responsiveness. Alternatively, other biodiversity conservation nonprofit leaders outlined specific ways in which changing economic conditions (e.g., the recent recession) affect their organizations:

Individual B (1): We manage an endowment worth about a half million dollars. We just try to manage the endowment sustainably. There is nothing built into our bylaws to take economic conditions into account for awarding money. During the recession, we have watched the money we have to work with decline due to the stock market. So that's made us look harder at how much to give out. Individual C (1): There is a bunch of ways the economy influenced what we do. We work really closely with local government officials. When housing developments happen locally, “over the transom” properties feed into the land trust. It is not planned, it is not strategic, and those properties have really variable biodiversity value. So for us, when the recession hit, housing development just stopped, and our vein of acquisition just stopped. Everything just shut down, and it is only beginning to start backup again. Individual D (1): Poor economic conditions tend to affect land trusts in two ways: we transition our fundraising to target wealthier individuals who are more insulated from tough economic times, and land gets cheaper. Because land is cheaper in a bad economy, a land trust can really get more bang for their buck. During the recession, there was plenty of opportunity for us to go after land we wanted, but the size of the projects we were able to do generally got smaller. The poor economy created both opportunities to acquire land and some constraints on our fundraising.

The preceding quotes show how changing (and specifically worsening) economic conditions can affect biodiversity conservation nonprofits by reducing endowments owing to stock market declines (B1), outright cessation of primary organizational activities (C1), and changing both funding and conservation opportunities, potentially in complex or contrasting ways (D1). This latter point (D1) seemingly relates to our main text results for TNC, in which this land trust was able to increase the proportion of conservation easements it acquired at below market value in poorer economic times. We attribute this as “donations” in the main text, but note here that this signal may represent a land market that changed in a worsening economy (i.e., owners increasingly willing to transact deals below fair market value).

### Appendix 4b

Major economic events and changing economic conditions do likely affect most biodiversity conservation nonprofits, as outlined by the preceding quotes (Section 4a) and hypothesized by our main text and preceding researchers (e.g., Bakker et al. 2010). Yet how do these organizations respond? As proposed in our financial ratio analysis, do biodiversity conservation nonprofits attempt to pursue strategic responses like growing liquid assets under good conditions; diversifying revenue streams; or cutting personnel costs under poor conditions? And are there meaningful repercussions if organizations neglect such strategies?

Individual A (2): In theory, we approach these issues pretty strategically. We did do an organizational risk assessment and flagged a major recession as the biggest threat to us. And our recession strategy was to diversify our revenue streams. That was the plan. But in practice, we are always desperate for money; we are always looking for everything we can get. We are very opportunistic. If the public funds shut off, then we go somewhere else. We will go to charitable trusts for example or seek contract income for services. But it has been instinct, not strategy. ‘Strategy’ is just market conditions cascading onto an organization. Individual B (2): We formed a committee and did an analysis on what would be sustainable for our endowment, and it is a running average of awarding about 4.5% of the endowment in grants per year. That is about $20k per year for us. We have gone just over that the last few years. When the stock market was booming, we were awarding more money each year. But recently, watching the endowment totals, they have been going down every year, so we are trying to make a slight adjustment. Individual C (2): Before the downturn, when I joined the board, I really wanted us to be more strategic and more proactive. So we found a property in a strategic way that we wanted to go after, and we fundraised deliberately for it, and we bought it. And that entire project straddles the pre- to post-recession divide. If you were an external observer looking at our organization, you might think we had done something strategic in response to the recession. The “over the transom” properties ceased, so we went out and bought something else. But it was really a decision we had made before the recession and independent of the recession. The transom properties just happened to cease right as we were deliberately trying to do something big and atypical for us. Individual D (2): I think recent economic challenges have created a transitional period for the land trust sector in general. Through most of our history, land trusts have acquired lands or acquired conservation easements and then flipped them onto public agencies for long-term management. Because of the economy and budget problems for governments, that does not happen nearly as much now. And it means that land trusts are having to think about stewardship, maintaining our own lands, more and more. There is a big difference between capital accumulation and stewardship. Stewardship is this ongoing cost associated with each property that the economy pushed onto us because public agencies can no longer fill that role. It's created a quandary for land trusts, because donors are more into acquiring land than managing it. Donors are not always interested in building trails, or thinning forest to promote old growth, or prescribed fire. It creates a real fundraising challenge for us. And it matters regardless of what happens in the future. If we cannot find an agency to take these lands, then they are ours to maintain indefinitely. And if at some point in the future a public agency does want to take these lands, we have to have maintained them so that they are in a condition that the agency is still interested in them.

Many of these preceding quotes interface in interesting ways with predictions from our financial ratio analysis. Our expectation that organizations should grow revenue sources (which requires considerable upfront costs; see A3 below) during good times is supported by A2 as a prerecessionary strategy. Our prediction that organizations (and especially larger organizations) might cut personnel costs as a means of buffering programmatic offerings during poor economic conditions is supported by E1, although this organization's subsequent growth through the recession suggests this deliberate strategy was premature. Further, D2 highlights reasons why growing liquid assets during favorable economic times may be prudent.

A land trust acquiring properties with no intention of retaining them for management, owing to typical transfer to government agencies, would not anticipate a need to grow a “stewardship endowment” to maintain these properties indefinitely (i.e., growing liquid assets). Under a changed economic reality in which government agencies are unable to assume management of these properties, the same land trust can find itself without the financial resources — and fundraising capacity to develop the financial resources — to manage these properties indefinitely. We would urge biodiversity conservation nonprofits to apply foresight in developing financial assets (i.e., growing liquid assets) proportional to potential future need during good economic conditions, even at the cost of some desired or possible conservation activity (i.e., land acquisition itself).

Alternatively, the preceding quotes also emphasize ways in which these organizations are not able to respond strategically — even if desired — to changing economic conditions. A2 reports that their organization aspires to be strategic in theory, but is generally opportunistic in practice (i.e., always looking for any available funding) regardless of economic conditions — a scenario we would anticipate is common to many biodiversity conservation nonprofits. B2 is a small organization that is responsive but not particularly strategic with respect to economic conditions; they award less of their endowment to fund research when economic conditions are poor, but generally aspire to a similar level of activity from year to year. C2 relates an instance in which a novel strategic land acquisition by their trust just happened to coincide with a major economic change, but was not precipitated by the change itself. In this case, an activity planned and initiated prior to a change in economic conditions was not enacted until after a major economic perturbation, demonstrating one way in which time lags may obscure or confuse responsiveness of biodiversity conservation nonprofits to changing economic conditions (see also E1 above). We discuss such lags, and their implications for our analyses and management of biodiversity conservation nonprofits, at some length in the main text.

We conclude with a brief selection of quotes that were surprising or unexpected with respect to how biodiversity conservation nonprofits were affected by, or responded to, changing economic conditions:

Individual A (3): We identified membership as an important area to grow, before the recession and independent of the recession. But it costs money. To grow membership, you have to employ people and you have to ask a lot of people. We used an external consultancy, and they charge commission for new membership. They were very successful for us in growing our membership, but lots of these external consultancies went bust during the recession. They had a kind of bad financial model. Because they operated on commission, during the recession, they had to ask more people to join an organization to get the same number to join as before, so they were doing more work with less income. Our external consultancy went bust in 2010. Incidentally, we actually went out and hired a bunch of their employees. We just brought them in-house and developed that capacity. And we are doing better now! Our profit from membership recruitment has gone up since the recession. Individual D (3): Something else that is really important is that when economic times are bad, we collaborate and leverage resources with other organizations more so than when times are good. It is not that we do not collaborate when times are good, but those collaborations are more strategic and more carefully defined. When the economy is bad, we are really willing to collaborate much more broadly and opportunistically.

A3 relates a fascinating example in which a potential hardship induced by poor economic conditions (a hired consultancy went bankrupt) ultimately resulted in an efficiency gain for the organization, in which they were able to hire some of the consultancy's previous employees and subsequently lower costs associated with fundraising by membership. Finally, D3 relates just one aspect of responsiveness to changing economic conditions that our main text analyses cannot account for: context-dependent patterns of collaboration and cooperation between organizations (e.g., Bode et al. 2010) in response to changing economic conditions. We highlight this example simply to emphasize that our efforts at characterizing responses of biodiversity conservation nonprofits to changing economic conditions are not intended as the complete story, but rather a starting point for any number of such investigations into organizational behavior (and hopefully organizational effectiveness) that could be pursued.

**Table A1:** Results of linear regression models for financial ratios of 90 biodiversity conservation nonprofits after detrending each response and GDP by time, and including organization as a random effect. Results are given for models excluding and including a term for interaction between nonprofit size and GDP. Sample selection bias has been simulated by excluding three small and two medium organizations with the largest loss in assets between 2000 and 2009 (Fig.2). The percent change in parameter estimates relative to the full model (Table2) is also given

	Size (SE); %	GDP (SE); %	Size × GDP (SE); %
No interaction			
Liquid funds interval	0.180 (0.030); −0.21	2.827 (1.218); −4.99	–
Revenue concentration	−0.088 (0.024); −0.15	−2.282 (0.787); 1.20	–
Personnel:Expenditures	0.042 (0.015); −0.01	−1.432 (0.480); −3.27	–
Liabilities:Assets	0.017 (0.017); −1.54	0.480 (0.741); −21.65	–
Interaction			
Liquid funds interval	0.180 (0.03); −0.21	7.495 (5.256); −7.99	−0.787 (0.863); −9.98
Revenue concentration	−0.88 (0.024); −0.12	11.581 (3.362); −0.45	−2.339 (0.552); −0.35
Personnel:Expenditures	0.042 (0.015); 0.01	−4.825 (2.069); −1.54	0.572 (0.034); −0.98
Liabilities:Assets	0.017 (0.017); −1.52	−1.597 (3.200); 29.29	0.351 (0.525); 17.38

**Table A2:** Results of linear regression models for financial ratios of 90 biodiversity conservation nonprofits after detrending each response and annual revenues by time, and including organization as a random effect. Results are given for models excluding and including a term for interaction between nonprofit size and annual revenue

	Size (SE)	Revenue (SE)	Size × Rev (SE)	Pseudo- *R* ^2^
No interaction				
Liquid funds interval	0.181 (0.030)***	0.031 (0.032)	–	0.204
Revenue concentration	−0.088 (0.024)**	−0.028 (0.020)	–	0.098
Personnel to total expend	0.042 (0.015)**	0.017 (0.012)	–	0.060
Liabilities to assets	0.017 (0.017)	−0.011 (0.019)	–	0.007
Interaction				
Liquid funds interval	0.181 (0.184)***	0.133 (0.093)	−0.018 (0.015)	0.204
Revenue concentration	−0.088 (0.024)**	0.115 (0.059)	−0.025 (0.010)*	0.100
Personnel to total expend	0.042 (0.015)**	0.123 (0.036)**	−0.019 (0.006)*	0.063
Liabilities to assets	0.017 (0.017)	−0.019 (0.056)	0.001 (0.009)	0.007

Significance of coefficients is given as ≤0.001 (***), ≤0.01 (**), and ≤0.05 (*). Pseudo- *R* ^2^ is given as the relationship of model fitted to observed response values.

**Table A3:** Results of linear regression models for financial ratios of 90 biodiversity conservation nonprofits after detrending each response and GDP by time, and including organization as a random effect. These models also evaluate whether geometric growth in revenue over the time period (2000–2009) for each biodiversity conservation nonprofit affects their financial behaviors as manifested by financial ratios. As in the main text, results are given for models excluding and including a term for interaction between nonprofit size and GDP

	Size (SE)	GDP (SE)	Size × GDP (SE)	Revenue (SE)	Pseudo- *R* ^2^
No interaction					
Liquid funds interval	0.181 (0.030)***	2.968 (1.211)*	–	0.008 (0.213)	0.206
Revenue concentration	−0.089 (0.024)**	−2.255 (0.780)**	–	−0.175 (0.171)	0.108
Personnel to total expend	0.042 (0.015)**	−1.479 (0.478)**	–	0.030 (0.109)	0.063
Liabilities to assets	0.017 (0.017)	0.0584 (0.737)	–	0.044 (0.119)	0.008
Interaction					
Liquid funds interval	0.181 (0.030)***	8.093 (5.230)	−0.866 (0.860)	0.008 (0.213)	0.206
Revenue concentration	−0.088 (0.024)***	11.634 (3.334)***	−2.347 (0.548)***	−0.175 (0.171)	0.113
Personnel to total expend	0.042 (0.016)**	−4.899 (2.063)*	0.578 (0.339)	0.030 (0.109)	0.063
Liabilities to assets	0.017 (0.017)	−1.130 (3.186)	0.290 (0.524)	0.044 (0.119)	0.008

Significance of coefficients is given as ≤0.001 (***), ≤0.01 (**), and ≤0.05 (*). Pseudo- *R* ^2^ is given as the relationship of model fitted to observed response values.
